# Complete Genome Sequence of *Caulobacter crescentus* Podophage Percy

**DOI:** 10.1128/genomeA.01373-15

**Published:** 2015-11-25

**Authors:** Roxann A. Lerma, T. J. Tidwell, Jesse L. Cahill, Eric S. Rasche, Gabriel F. Kuty Everett

**Affiliations:** Center for Phage Technology, Texas A&M University, College Station, Texas, USA

## Abstract

Podophage Percy infects *Caulobacter crescentus*, a Gram-negative bacterium that divides asymmetrically and is a commonly used model organism to study the cell cycle, asymmetric cell division, and cell differentiation. Here, we announce the sequence and annotated complete genome of the phiKMV-like podophage Percy and note its prominent features.

## GENOME ANNOUNCEMENT

*Caulobacter crescentus* is a nonpathogenic bacterium that is found in diverse environments, especially fresh water lakes and soil (1). Cell division in *C. crescentus* is asymmetrical and yields two types of cells that are structurally and functionally different: swarmer cells and stalked cells (2). *Caulobacter* phages have been used to study host genetic regulation and morphogenesis via generalized transduction, but typically these are large, virulent myophages or siphophages with prolate capsids (3–5). Here, we describe the complete genome of Percy, a newly isolated phiKMV-like podophage which infects *C. crescentus* strain CB15.

Bacteriophage Percy was isolated from a water sample collected in College Station, Texas, USA. Phage DNA was sequenced using 454 pyrosequencing at the Emory GRA Genome Center (Emory University, GA, USA). Trimmed FLX Titanium reads were assembled to a single contig at 41.7-fold coverage using the Newbler assembler version 2.5.3 (454 Life Sciences) at default settings. The contig was confirmed to be complete by PCR using primers that face the upstream and downstream ends of the contig. Products from the PCR amplification of the junctions of concatemeric molecules were sequenced by Sanger sequencing (Eton Bioscience, San Diego, CA, USA). Genes were predicted using GeneMarkS (6) and corrected using software tools available on the Center for Phage Technology (CPT) Galaxy instance (https://cpt.tamu.edu/galaxy-public). Morphology was determined using transmission electron microscopy performed at the Texas A&M University Microscopy and Imaging Center.

Percy has a 44,773-bp unit genome (does not include the terminal repeat), a coding density of 94%, 55 predicted coding sequences, and a G+C content of 60.9%. Of the 55 predicted coding sequences, 21 are novel, 9 are conserved, and 25 have an annotated gene function predicted by BLASTp, InterPro Scan, and CDD analyses (7–9). The G+C content of the phage is lower than that of its host (67.2%) (10). A 201-bp terminal repeat was determined using the PAUSE method on raw sequencing data (https://cpt.tamu.edu/pause), resulting in a packaged genome of 44,974 bp. Percy shares 49.7% and 53.8% nucleotide sequence identity across the genome with *Pseudomonas* phage phiKMV (NC_005045) and *Caulobacter* phage Cd1 (GU393987), respectively, as determined by Emboss Stretcher (11). Core gene analysis shows that Percy and Cd1 share 66% homology (31 out of 47 genes) (12). Most of the differences between the two phages are in hypothetical proteins of unknown function.

As a phiKMV-like phage, Percy encodes a T3/T7-like RNA polymerase that is downstream from the replication genes rather than in the early gene region, as is the case in *Enterobacteria* phage T7 (NC_001604) (13). The DNA ligase of Percy is located upstream of the RNA polymerase and is out of place compared to most phiKMV-like phages (14). Percy also encodes a phiKMV-like internal head protein with a predicted C-terminal lysozyme domain that presumably plays a role in infection as an injection needle component (15, 16). To accomplish lysis, Percy encodes a pinholin, signal anchor release endolysin, and partially imbedded spanin genes (17–19).

### Nucleotide sequence accession number.

The genome sequence of phage Percy was deposited in GenBank under the accession number KT381879.
